# Limnological effects of a large Amazonian run-of-river dam on the main river and drowned tributary valleys

**DOI:** 10.1038/s41598-019-53060-1

**Published:** 2019-11-14

**Authors:** Rafael M. Almeida, Stephen K. Hamilton, Emma J. Rosi, João Durval Arantes, Nathan Barros, Gina Boemer, Anderson Gripp, Vera L. M. Huszar, Pedro C. Junger, Michele Lima, Felipe Pacheco, Dario Carvalho, Alexander J. Reisinger, Lúcia H. S. Silva, Fábio Roland

**Affiliations:** 1 Department of Biology, Federal University of Juiz de Fora, Juiz de Fora, MG Brazil; 2 Cary Institute of Ecosystem Studies, Millbrook, NY USA; 3 W.K. Kellogg Biological Station and Department of Integrative Biology, Michigan State University, Hickory Corners, MI USA; 4 Ecology and Environment do Brasil Ltda., Rio de Janeiro, RJ Brazil; 5 Institute of Biodiversity and Sustainability, Federal University of Rio de Janeiro, Rio de Janeiro, RJ Brazil; 6 National Museum, Federal University of Rio de Janeiro, Rio de Janeiro, RJ Brazil; 7 Department of Hydrobiology, Federal University of São Carlos, São Carlos, SP Brazil; 8 Earth System Science Center, National Institute for Space Research, São José dos Campos, SP Brazil; 9 Santo Antônio Energia, Porto Velho, RO Brazil; 10 Soil and Water Sciences Department, University of Florida, Gainesville, FL USA; 11 Present Address: Department of Ecology and Evolutionary Biology, Cornell University, Ithaca, NY USA

**Keywords:** Environmental impact, Limnology

## Abstract

Run-of-river dams are often considered to have lower environmental impacts than storage dams due to their smaller reservoirs and low potential for flow alteration. However, this has been questioned for projects recently built on large rivers around the world. Two of the world’s largest run-of-river dams—Santo Antônio and Jirau—were recently constructed on the Madeira River, a major tributary to the Amazon River in Brazil. Here we evaluate the effects of the creation of the Santo Antônio dam on the water chemistry and thermal structure of the Madeira River mainstem and back-flooded valleys of tributaries within the reservoir inundated area. In contrast to the mainstem river, some back-flooded tributaries periodically developed thermal stratification, which is associated with higher water residence times. Additionally, biochemical oxygen demand, partial pressure of CO_2_, and organic carbon all increased in the tributary valleys inundated by the reservoir, possibly due to increased input of allochthonous organic matter and its subsequent mineralization upon back-flooding—a common feature of newly flooded impoundments. The mainstem did not show detectable dam-related changes in water chemistry and thermal structure. Although the majority of the reservoir area maintained riverine conditions, the lateral valleys formed upon back-flooding—corresponding to ~30% of the Santo Antônio reservoir area—developed lake-like conditions akin to a typical reservoir of a storage dam.

## Introduction

Run-of-river hydropower plants have smaller reservoirs with limited water storage potential, unlike storage dams, which generally form large reservoirs with lacustrine conditions and variable water volumes. Inflowing water typically passes through a run-of-river reservoir quickly, and the electricity generation is a function of the flow of the river at a given time. Run-of-river dams are often considered to have lower environmental impacts due to their smaller reservoirs and lower potential for flow alteration^1,2^, although this has been questioned for projects recently built on large rivers around the world^3,4^. Considering that most new dam construction is of the run-of-river design^5^, there is an urgent need to more fully document the effects of this dam design on river systems.

Two of the world’s largest run-of-river dams—Santo Antônio and Jirau—have recently been constructed on the Madeira River, the largest tributary to the Amazon River in terms of water and sediment discharge^6,7^. The Jirau dam is immediately upstream of the reservoir created by the Santo Antônio dam, and the combined installed capacity of these two projects is equivalent to ~30% of the installed capacity of China’s Three Gorges dam, the largest in the world. Unlike some smaller run-of-river projects, these two large dams created reservoirs, inundating a total of ~900 km^2^ ^8^. The Santo Antônio and Jirau dams were built so that, after being flooded, the reservoir is kept at bankfull stage throughout the year, with very little variation in the water level (70.5 to 71.3 m a.s.l. at Santo Antônio).

The Madeira dams were approved for construction after a great deal of controversy^9^. The Madeira River carries large amounts of suspended sediments that control downstream geomorphology^10^ and support highly productive fringing floodplains^7^. For these reasons, there has been much concern regarding how the Madeira dams could affect the natural flow of sediments and associated nutrients in the Madeira River^10–12^. Now, a few years after the dams were built, studies are starting to reveal decreases in downstream suspended sediment concentrations and fisheries yields^13–15^. These new studies have focused on downstream impacts, but the extent to which the dam has modified upstream water chemistry and thermal structure, and how the changes affect the biota of the newly formed aquatic environments, is not known. Consideration of upstream impacts is especially important considering that remote sensing has revealed that the flooded area estimated in the pre-dam environmental impact assessment may have been ~60% too low^8^, which may be in part related to changes in project design—reservoir level is currently 80 cm higher than that considered in pre-dam assessments.

Considering that the vast majority of the hydropower dams constructed in Brazil over the past decade have been of the run-of-river design, purportedly to minimize adverse ecological impacts^16,17^, a solid understanding of the ecological effects of these projects is crucial to inform future planning and management strategies. This is especially true considering the expected proliferation of hydropower dams of the run-of-river type throughout the Amazon basin^18,19,20^. Here, we evaluate the upstream effects of the creation of the Santo Antônio reservoir on the water chemistry and thermal structure of the Madeira River mainstem and its back-flooded tributaries. We compare pre- and post-dam patterns of ten ecologically and biogeochemically relevant variables: conductivity, turbidity, total phosphorus, total organic carbon, dissolved organic carbon, dissolved inorganic carbon, pH, partial pressure of CO_2_ (pCO_2_), dissolved oxygen, and biochemical oxygen demand (BOD). We sampled stations in the mainstem river and in back-flooded tributary valleys; because the back-flooded tributaries have longer water residence times^21,22^, we hypothesized that they would display more marked changes in water quality.

## Results

### Water depth

**Figure 1:**
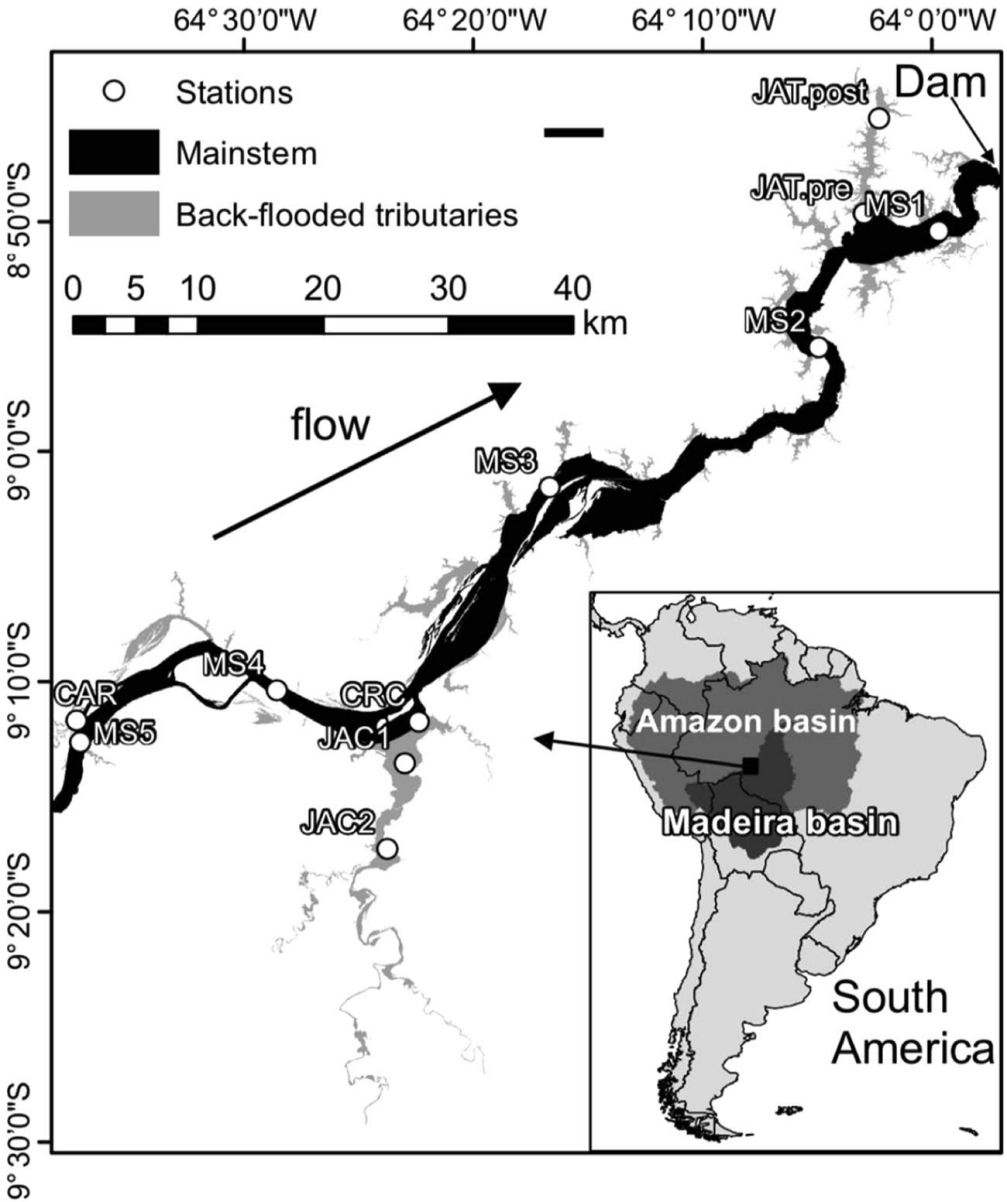
Map of the Santo Antônio reservoir, with black and grey areas depicting the area covered by the mainstem (70%) and back-flooded tributary valleys (30%), respectively, based on a total inundated area of 471 km^2^. Our sampling station in the tributary JAT was moved upstream after damming. Figure adapted from^42^.

Analysis of water depths before and after damming indicates that the dam shifted both the mainstem and tributaries in its area of influence to a permanent high-water condition. The lower reaches of the tributaries that flow into the Madeira River within the reservoir area were permanently back-flooded upon damming, forming lateral drowned valleys that account for nearly 30% of the reservoir area (Fig. 1). In the mainstem, the average depth increased from 10 ± 3 (standard deviation) m to 22 ± 8 m during low-water phases (Student’s *t*-test, *t* = −4.2, df = 18, *P* < 0.05), but there was not a significant difference between pre- (21 ± 5 m) and post-dam (24 ± 6 m) depths during high-water phases (Student’s *t*-test, *t* = 1.2, df = 18, *P* = 0.23). The same occurred in the back-flooded tributaries, where depth increased from 1.3 ± 0.7 m (pre-dam) to 6.9 ± 0.8 m (post-dam) during low water phases (Student’s *t*-test, *t* = −15.9, df = 18, *P < *0.05), but not during high-water phases (9.3 ± 1.2 m pre-dam versus 8.9 ± 1.5 m post-dam) (Student’s *t*-test, *t* = 0.7, df = 18, *P* = 0.49).

### Thermal profiles

**Figure 2:**
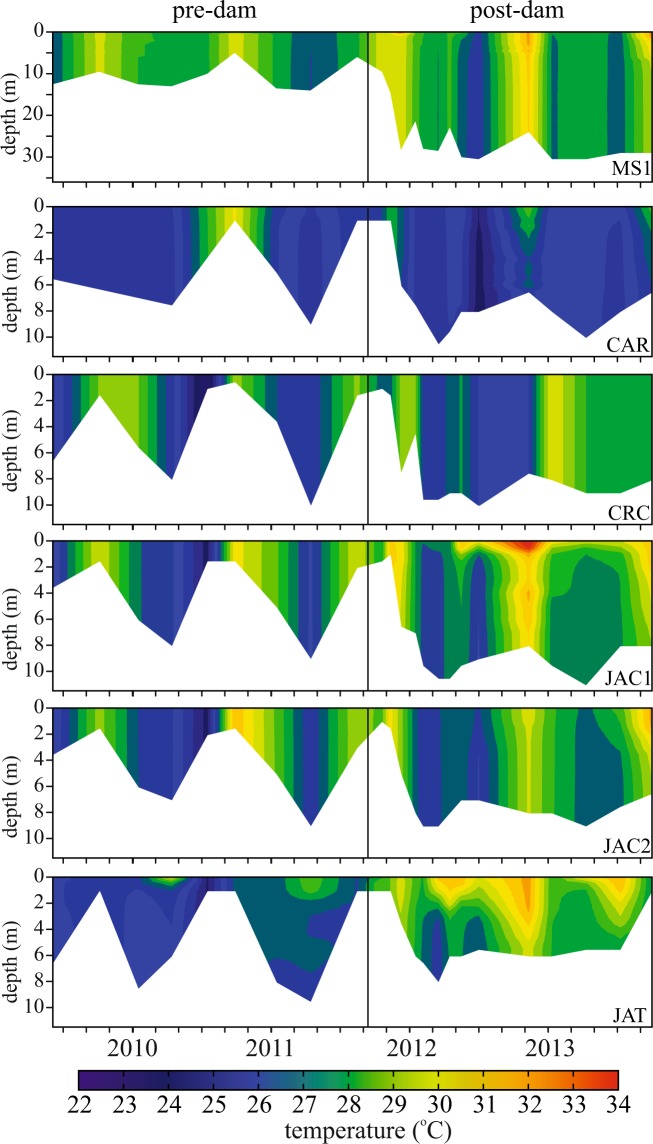
Depth-time isotherms (°C) in the mainstem (MS1) and in back-flooded valleys of tributaries (CAR, CRC, JAC1, JAC2, and JAT) within the Santo Antônio reservoir before and after damming. The vertical black line indicates dam closure. Note that the JAT station was moved further upstream after damming (see Fig. 1). Figure adapted from^42^.

The dam resulted in the formation of lacustrine conditions in some of the back-flooded tributaries, whereas lotic conditions were maintained in the mainstem. Two of the back-flooded tributary valleys occasionally developed thermal stratification after damming, whereas the water column in the mainstem remained isothermal as before (Fig. 2). The stratification of the back-flooded tributaries is a response to the increased water residence time caused by dam closure. The tributary valley that was most strongly stratified (JAT) has the highest residence time in the reservoir area (up to 36 days). In the mainstem, the post-dam residence time was short (mean: 2.4 days) and the water flow was too fast (mean: 0.33 m s^−1^) to allow thermal stratification.

### Water chemistry

**Figure 3:**
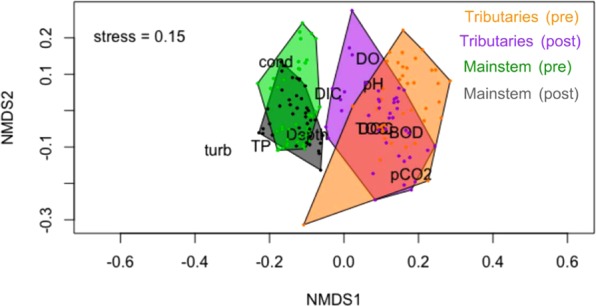
Non-metric multidimensional scaling (NMDS) ordination showing the clustering in water chemistry between the mainstem and back-flooded tributary samples from the Santo Antônio reservoir before and after dam closure. There was no pre- versus post-dam difference in the mainstem (PERMANOVA, r^2^ = 0.01, *P* = 0.34), but a significant difference was observed in the back-flooded tributaries (PERMANOVA, r^2^ = 0.07, *P* < 0.05). Figure adapted from^42^.

The overall water chemistry changed substantially after damming in the back-flooded tributary valleys, but not in the mainstem (Fig. 3). A PERMANOVA analysis indicated that the overall water chemistry of the mainstem did not significantly differ before and after dam closure (r^2^ = 0.01, *P* = 0.34), whereas a significant difference was observed in the tributaries (r^2^ = 0.07, *P* < 0.05). After dam closure, the back-flooded valleys of the tributaries became chemically more similar to the Madeira River, which is illustrated by the NMDS polygon with post-dam tributary samples moving towards the mainstem polygons— although their lower conductivity shows that they remained relatively diluted in total ionic content, as is characteristic of lowland Amazonian rivers compared to those of Andean origin (Fig. 3). The “stress” of the NMDS was <0.20, which indicates good representation for ecological data.

**Figure 4:**
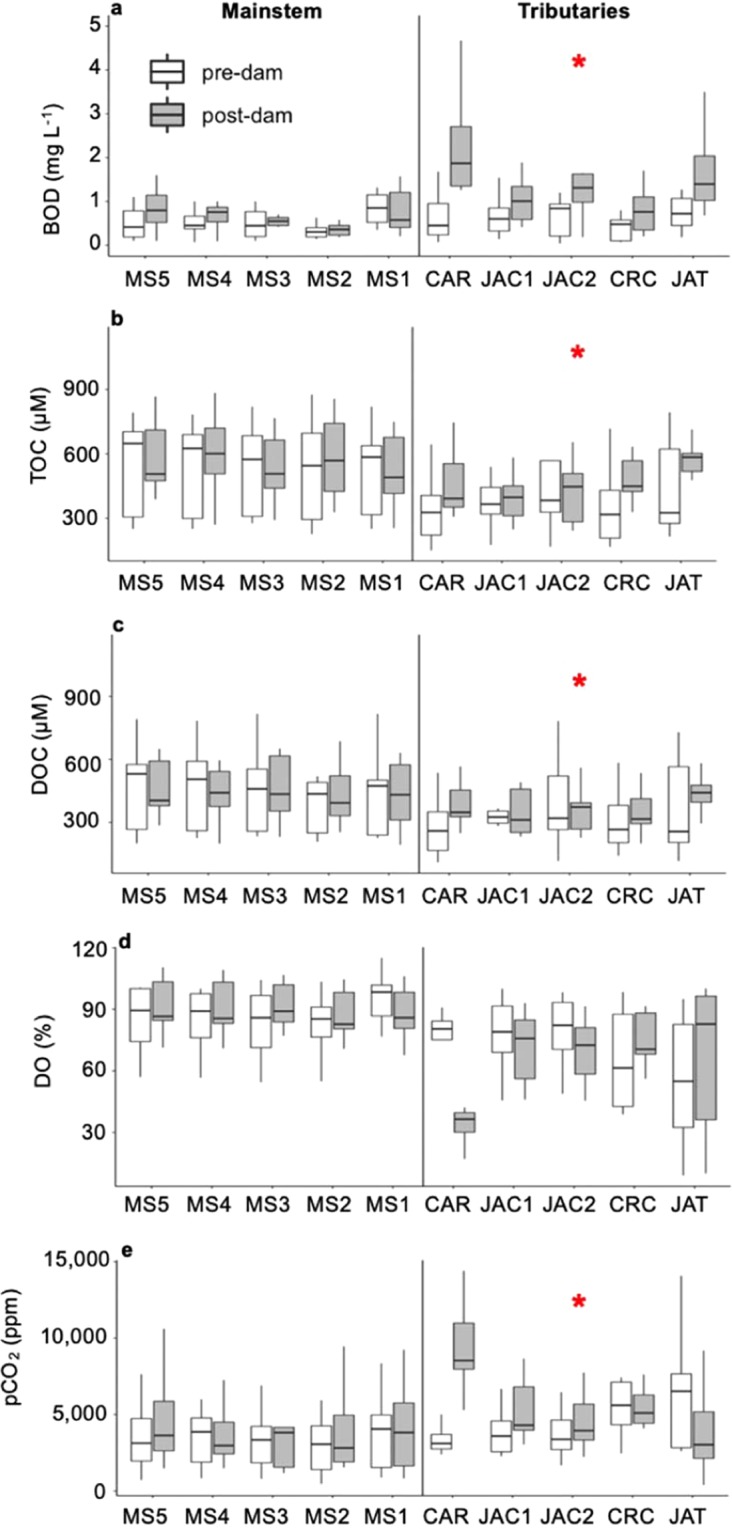
Box-plots depicting pre- (white) and post-dam (grey) comparisons of (**a**) biochemical oxygen demand (BOD); (**b**) total organic carbon (TOC) concentrations; (**c**) dissolved organic carbon (DOC) concentrations; (**d**) dissolved oxygen percent saturation (DO); and (**e**) partial pressure of CO_2_ (pCO_2_). The graphs in the first column are for the five mainstem stations and the graphs in the second column depict results for the five stations in back-flooded valleys of the tributaries. The red stars indicate significant differences between pre- and post-dam periods (p < 0.05). The horizontal line inside the box indicates the median and the boundaries indicate the 25^th^ and 75^th^ percentiles. Outliers are not shown for improved visualization.

**Figure 5:**
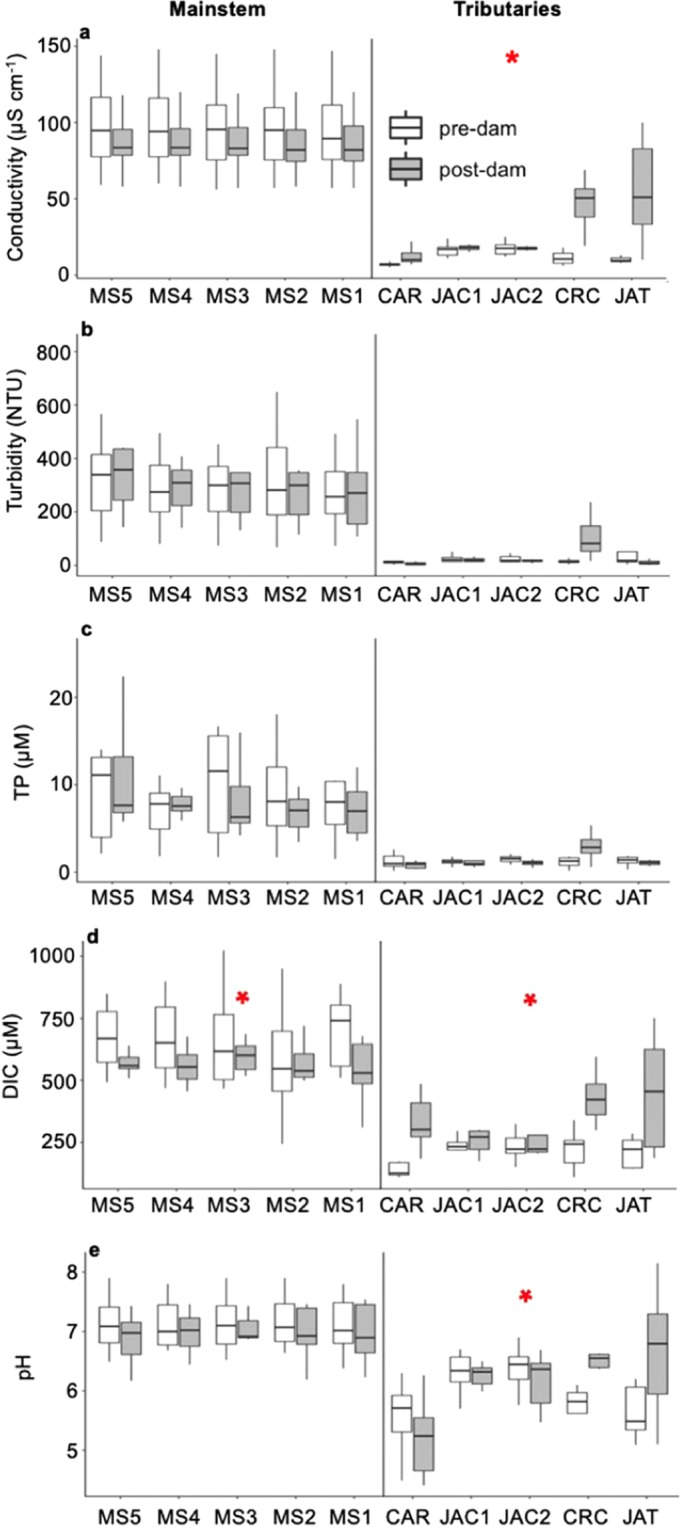
Box-plots depicting pre- (white) and post-dam (grey) comparisons of (**a**) electrical conductivity (EC); (**b**) pH; (**c**) turbidity; (**d**) total phosphorus (total P); and (**e**) dissolved inorganic carbon (DIC) concentrations. The graphs in the first column are for the five mainstem stations and the graphs in the second column depict results for the five stations in the back-flooded valleys of the tributaries. The red stars indicate significant differences between pre- and post-dam periods (p < 0.05). The horizontal line inside the box indicates the median and the boundaries indicate the 25^th^ and 75^th^ percentiles. Outliers are not shown for improved visualization.

In the mainstem, we found that the concentrations of dissolved inorganic carbon decreased significantly after damming, but we could not find significant alterations for BOD, total organic carbon, dissolved organic carbon, dissolved oxygen, pCO_2_, electrical conductivity, turbidity, total phosphorus, and pH (Figs 4 and 5). Prior to damming, the downstream-most mainstem station (MS1) had higher oxygen concentrations than the others, as there was a waterfall between MS2 and MS1. With reservoir creation, the waterfall was inundated and DO concentrations became longitudinally homogenous along the reservoir mainstem.

In the back-flooded valleys of the tributaries, dissolved oxygen, turbidity, total phosphorus, and pH did not differ between pre- and post-dam samplings, but we found that BOD, total organic carbon, dissolved organic carbon, pCO_2_, electrical conductivity, and dissolved inorganic carbon all increased significantly after damming (Figs 4 and 5). DO showed a peculiar pattern in the tributaries, with decreases in CAR, JAC1 and JAC2 being counterbalanced by increases in CRC and JAT, whereas the opposite trend was observed for pH and pCO_2_ (Fig. 4).

**Figure 6:**
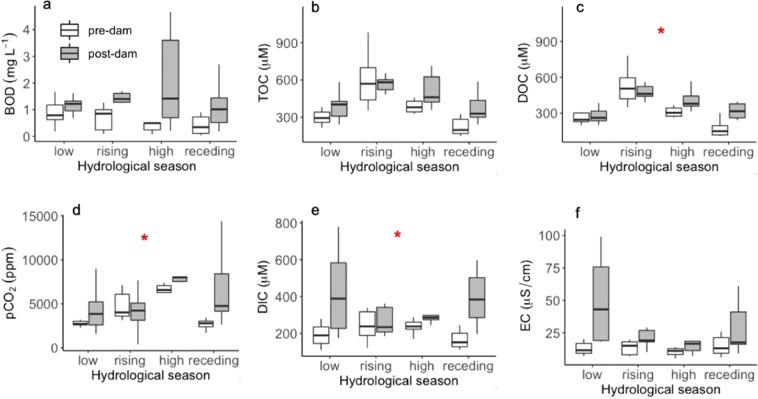
Box-plots depicting pre- (white) and post-dam (grey) comparisons of biochemical oxygen demand (BOD; **a**) total organic carbon (TOC; **b**) dissolved organic carbon (DOC; **c**) partial pressure of dissolved CO_2_ (pCO_2_; **d**) dissolved inorganic carbon (DIC; **e**) and electrical conductivity (EC; **f**) for each of the four phases of the hydrological cycle in the five stations located in back-flooded valleys of the tributaries (CAR, JAC1, JAC2, CRC, and JAT). The red stars indicate significant interactions (p < 0.05) between project stage (pre- and post-dam) and hydrological season (low, rising high, and receding waters) for DOC, pCO_2_, and DIC. The horizontal line inside the box indicates the median and the boundaries indicate the 25^th^ and 75^th^ percentiles. Outliers are not shown for improved visualization.

For variables displaying significant pre- versus post-dam differences in the back-flooded valleys of the tributaries, we further analyzed interaction between hydrological season (i.e., low, rising, high, and receding waters) and project stage (pre- and post-dam). We found that differences between pre- and post-dam periods occurred regardless of the hydrological season for BOD, TOC and conductivity (Fig. 6). For DOC, pCO_2_ and DIC, pre- and post-dam differences depended on the hydrological season, with rising-water periods not presenting pre- versus post-dam differences (Fig. 6).

## Discussion

This study investigated the upstream effects of the creation of the Santo Antônio reservoir on the water chemistry and thermal structure of the Madeira River mainstem and tributary valleys flooded by the reservoir. We found minimal effects on the mainstem of the river and more pronounced effects on the lateral valleys. Impoundments typically change the condition of the water body upstream of the dam from lotic to lentic, increasing the water residence time. This hydrological change affects physical, chemical, and biological characteristics of the water body, often leading to thermal stratification and modifications in biogeochemical cycles^23^. The water slowdown may increase particle settling, decreasing turbidity and nutrient concentrations^24,25^. Dissolved oxygen concentrations can decrease, and CO_2_ production can increase following organic matter enrichment. None of these changes generally observed after damming was observed in the mainstem of the Santo Antônio reservoir, which accounts for ~70% of the reservoir surface area. However, dam-induced changes were observed in the lateral valleys formed by back-flooded tributaries (30% of the reservoir surface area), where many variables exhibited significant changes in pre- to post-dam comparisons (Figs 4 and 5). Our findings suggest that, in reservoirs of some run-of-river dams, even though the mainstem may largely maintain river-like conditions, the lateral drowned tributary valleys formed upon back-flooding may develop lake-like conditions akin to a typical reservoir of a storage dam. These changes in back-flooded valleys of tributaries are likely associated with their higher water residence times, which have been shown to be strongly correlated with a variety of functional metrics in both lotic and lentic ecosystems such as organic matter decomposition and chemistry^26^, internal metabolism^27^ and hypoxia development^28^, and nutrient uptake and delivery rates^29^.

We could not detect a significant reduction in turbidity or in total phosphorus, which is primarily particle-bound in the Madeira River^11^, within the Santo Antônio reservoir. This is probably because the water residence time is short, precluding the degree of particle sedimentation that is typically observed for large reservoirs of storage dams^25,30,31^. Two recent studies have found reductions of 20–30% in the concentrations of fine suspended sediments downstream of the Madeira dams^13,14^. However, a concomitant reduction of >30% was observed upstream of the dams^13^, which suggests that it is not possible to attribute the changes in fine suspended sediment concentrations to the creation of the dams. It remains unclear to what extent the Madeira dams have altered the sediment dynamics of the Madeira River. Although near-surface results do not show detectable reductions in turbidity and fine suspended sediments^13^, we note that the dams may still be trapping coarser sediments.

Our results for the thermal structure of the Santo Antônio reservoir corroborate a modelling study that estimated the densimetric Froude number for the mainstem of the Santo Antônio reservoir^21^. The Froude number is a dimensionless number that indicates the likelihood that the main body of a reservoir will develop thermal stratification; Froude values above 1 indicate that thermal stratification is unlikely. Monthly Froude values of the mainstem of the Santo Antônio reservoir ranged from 10 (lowest discharge) to 62 (highest discharge)^21^. Indeed, we found a well-mixed water column in the mainstem. In addition, we found occasional thermal stratification in the back-flooded valleys of the tributaries, which is especially likely during low discharge^21^, when the water residence time increases and flushing rates decrease. Thermal stratification was stronger and more persistent in JAT (Fig. 2), the back-flooded tributary valley with the largest residence time^22^.

Dissolved inorganic carbon was the only mainstem variable that displayed significant change between pre- and post-dam comparisons (Fig. 5). Previously reported measurements made at Porto Velho over four pre-dam years (2004–2007) showed high interannual variability in concentrations of HCO_3_^−^, the primary component of dissolved inorganic carbon in these waters, with annual means ranging from 142–519 μM^32^. The difference we report between pre- and post-dam concentrations of dissolved inorganic carbon is well within this range of natural interannual variability (Fig. 5), and thus it is possible that the lower concentrations of dissolved inorganic carbon concentrations after damming are related to a naturally occurring variation among years. Our study does not provide evidence for major changes in the water chemistry of the mainstem that can be unambiguously attributed to damming of the river.

While the NMDS and pre- versus post-dam comparisons did not indicate a large effect of the dam on the water chemistry of the mainstem, there were substantial changes in the back-flooded tributaries (Figs 3, 4 and 5). We attribute the alterations observed in the back-flooded tributaries to two different causes. First, changes in conductivity and dissolved inorganic carbon after damming indicate that the back-flooded valleys of the tributaries still contained water of largely local origin but became chemically more similar to the mainstem (Figs 4 and 5); this is corroborated by the NMDS ordination showing that the polygon representing post-dam tributary samples moving towards the mainstem polygons (Fig. 3). Second, increases in BOD, pCO_2_, total organic carbon and dissolved organic carbon are likely a result of increased organic matter mineralization of terrestrial carbon upon back-flooding, as is common for the initial years after damming^33^. As a reservoir ages, the mineralization of organic matter typically slows down, as does CO_2_ production^34–36^, so it is likely that CO_2_ supersaturation will decrease over time as the newly created Santo Antônio reservoir ages. Concentrations of dissolved oxygen did not show a significant difference after damming, but the magnitude of the difference in median values is consistent with the increased CO_2_ from respiration of organic matter. Both DO and pCO_2_ varied distinctly when comparing pre- and post-dam measurements in different back-flooded tributary valleys (Fig. 4), which may be associated with different inundated landscapes^33^. In addition, changes in phytoplankton biomass among the back-flooded valleys of the tributaries may explain spatial variations in DO and pCO_2_^37^.

Amazonian reservoirs are typically large sources of carbon emissions to the atmosphere^34,38,20^. Our results suggest that the creation of the Santo Antônio reservoir did not result in a net increase of CO_2_ supersaturation in the mainstem. In three sampling stations in back-flooded valleys of the tributaries we found a consistent net increase in CO_2_ supersaturation after dam closure. This is consistent with the findings of a recent study indicating that the lateral drowned valleys of Amazonian reservoirs that have short overall water residence times are hot spots for CO_2_ emissions^21^, and also with findings suggesting that methane emissions from back-flooded tributaries of the Santo Antônio reservoir are substantially higher than from the mainstem^39,40^. Hence, our results build on previous work, reinforcing that while CO_2_ emissions may not increase in the mainstem of run-of-river Amazonian reservoirs^41^, they can be higher in relatively stagnant lateral valleys and back-flooded valleys of tributaries.

In summary, the lateral valleys created by back-flooding occupy about 30% of the Santo Antônio reservoir area. We found that this run-of-river dam had a larger effect on the water chemistry and thermal structure of back-flooded tributaries than on the mainstem river within the reservoir. We detected evidence of incursion of mainstem water into the back-flooded tributaries, as indicated by conductivity and dissolved inorganic carbon. In addition, these lateral valleys were more prone to developing thermal stratification due to residence times that can exceed 30 days^22^, unlike the mainstem that retained lotic conditions as is generally expected for dams of the run-of-river design. We also found that organic matter input and subsequent mineralization increased in the lateral valleys after damming, as indicated by higher concentrations of total organic carbon, dissolved organic carbon, BOD, and pCO_2_. It is likely that tributary waters flooding soils rich in organic matter rather than inputs via mainstem water are responsible for the observed increases in organic carbon, BOD, and pCO_2_ in the back-flooded valleys of tributaries, as these increases persisted during the majority of the hydrological cycle (Fig. 6). Because of their greater differences in physical and chemical characteristics, it is likely that back-flooded areas of run-of-river dams produce a different habitat for the aquatic biota compared to the mainstem waters, creating conditions that are favorable to freshwater species adapted to lentic environments^37^. Our study advances the scarce understanding of the environmental impacts of large run-of-river dams. We provide evidence that the lateral valleys potentially created by back-flooding should be considered priority areas when designing protocols for environmental impact assessments and monitoring programs for run-of-river dams.

## Methods

### Study site

The Santo Antônio dam is a run-of-river hydropower plant located on the Madeira River, in the state of Rondônia, Brazil (Fig. 1). The dam is 60-m tall and 2.5-km wide. The official area of the reservoir is 471 km^2^, although remote sensing indicates a larger flooded area^8^; throughout this study, we use the official reservoir area. The reservoir volume is 2075 × 10^6^ m^3^, the length of the reservoir is 130 km, and the average depth is 11 m. The total installed capacity of 3,568 MW is provided by 50 bulb turbines. The environmental licensing of the Santo Antônio dam was characterized by much controversy—particularly with respect to sediment accumulation in the reservoir—and a detailed overview is provided in^9^. The dam was completed in September 2011, and the average water residence time of the reservoir is 2.4 days^42^. The average velocity of the Madeira River at Porto Velho prior to damming was 1.3 m s^−1^ (SO-HyBAm, http://www.ore-hybam.org/), which indicates that the natural (pre-damming) time for the water to travel through the reservoir length was about one day. A hydrodynamic and water quality model (CE-QUAL-W2) developed during the environmental licensing of Santo Antônio dam^22^ indicated that water residence times in drowned tributary valleys are about five times larger than in the Madeira mainstem, reaching up to 35 days in the Jatuarana (JAT) valley (Fig. 1), the back-flooded valley with the highest residence time in the reservoir. Throughout the reservoir, residence times peak in the dry season, typically between August and October. The hydrodynamic model also estimated annual average discharges in two of the back-flooded tributaries, JAT (1 m^3^ s^−1^) and Jaci-Paraná (JAC, 165 m^3^ s^−1^).

The Madeira River basin drains parts of Bolivia, Peru, and Brazil. With an area of 1.4 × 10^6^ km^2^, it covers 23% of the Amazon basin and 35% of the Andean Amazon^43^. The Madeira River has the third largest suspended sediment load among tropical rivers, behind the Amazon and Brahmaputra rivers^6^. The Madeira River is the largest tributary to the Amazon River and the world’s fourth largest tropical river in terms of discharge^6^, contributing 15, 14, and 50% of the Amazon River’s water, organic carbon, and suspended sediment transport, respectively^6,44^. Due to its origin in the high Andes, the Madeira River is rich in sediments and associated nutrients, as opposed to the small tributaries that flow into the reservoir, which are clear-water systems draining older terrains. The climate of the Madeira River basin is humid tropical – Am in the Köppen classification^45^. The mean annual precipitation is ~2,000 mm^44^, which is unevenly distributed across the year^46^. Discharge in the Madeira River can vary by an order of magnitude between low and high flow months, averaging 19,000 m^3^ s^1^ downstream of the dam at Porto Velho^11^ and 31,000 m^3^ s^−1^ at the river’s confluence with the Amazon River^44^. The largest cities within the Madeira River basin are La Paz and Santa Cruz de la Sierra in Bolivia, and Porto Velho in Brazil. Together, these three cities comprise a population of ~3.5 million people; otherwise, the Madeira River basin is sparsely populated.

### Sampling and analytical procedures

We sampled five stations along the Madeira River mainstem within the reservoir and five stations in tributaries (Igarapé Caripuna, CAR; Jaci-Paraná River, JAC; Caracol River, CRC; Igarapé Jatuarana, JAT) that drain into the reservoir (Fig. 1); these tributaries were back-flooded upon damming and all our sampling stations are located within the reservoir inundated area. Sixteen field sampling campaigns were performed quarterly between 2009 and 2013, encompassing all major phases of the flooding cycle. The field campaigns were preferentially conducted in October (low water), January (rising water), April (high water), and June-July (receding water). Our study comprises eight field campaigns performed prior to damming (2009–2011) and eight field campaigns performed after damming (2012–2013). The filling of the reservoir started in September 2011 and it reached full pool in January 2012.

Measurements and samples were taken from ~0.5 m below the water surface. Water depth was measured at the time of sampling with a Garmin GPSmap sounder. Water temperature, conductivity, turbidity, dissolved oxygen and pH were measured at the time of sampling with a multiparameter sonde (YSI, model 6920) previously calibrated according to the manufacturer’s instructions. Five-day biochemical oxygen demand (BOD) measurements were made via incubations started within a day after sampling; dissolved oxygen was measured initially and after five days, and the BOD was computed from the difference between initial and final dissolved oxygen. Subsamples were filtered through GF/C glass-fiber filters (effective pore size ~0.7 μm) for analysis of DOC. Filtered and unfiltered water samples for laboratory analysis were kept refrigerated in the dark at 4 °C until analysis. The analyses were performed within a week of completion of each field campaign. Total phosphorus was measured by the colorimetric molybdate blue method^11^. Total organic carbon, dissolved inorganic carbon, and dissolved organic carbon were analyzed on a Tekmar-Dohrmann TC Analyzer (Model Phoenix 8000). Partial pressure of CO_2_ (pCO_2_) was calculated from measurements of pH and dissolved inorganic carbon^47,48^. In addition, we measured depth profiles of temperature to understand how the reservoir affected stratification and mixing in the mainstem and in back-flooded tributary valleys. Because all five mainstem stations remained vertically isothermal, we only present data for the station immediately above the dam.

### Data analysis

We first quantified changes in the overall water chemistry of the mainstem and back-flooded valleys of the tributaries in response to the dam closure. We used non-metric multidimensional scaling (NMDS) based on a Bray-Curtis distance to visualize clustering in water chemistry among the mainstem and tributary samplings before and after dam closure, followed by permutational multivariate analysis of variance (PERMANOVA) to test for differences among groups. Two PERMANOVAs were run, one for the mainstem (pre- vs. post-dam) and one for the tributaries (pre- vs. post-dam). Although NMDS is often used to analyze differences in community composition across sites, here we use our various response variables in place of species abundances, which would traditionally drive the NMDS. Both NMDS and PERMANOVA were completed with the *vegan* package, using the *metaMDS* and *adonis* functions, respectively^49^ in the R Statistical Software version 3.3.2^50^.

We used linear mixed-effects models to evaluate the effect of the dam on the response variables using the *lmer* function of the package *lme4*^51^ in the R software. We log-transformed response variables (except pH) to meet normality and homoscedasticity assumptions. For all response variables, sampling station was considered as a random effect to avoid problems associated with multiple pairwise testing^52^.

## Supplementary information

Supplementary Dataset 1

## Data Availability

Supplementary information is available for this paper (spreadsheet with raw data used for the analyses).
